# Prevalence of Brucellosis in Small Ruminants in Africa from 2000 to 2025: A Systematic Review and Meta-Analysis

**DOI:** 10.3390/vetsci13070638

**Published:** 2026-06-30

**Authors:** Weldeab Solomon Ghebrezgabher, Jiazhen Ge, Guodong Song, Fuying Zheng, Yuefeng Chu

**Affiliations:** 1 State Key Laboratory for Animal Disease Control and Prevention, College of Veterinary Medicine, Lanzhou University, Lanzhou Veterinary Research Institute, Chinese Academy of Agricultural Sciences, Lanzhou 730046, China; weldeabsolomon32@gmail.com (W.S.G.); 18256589168@163.com (J.G.); songguodong166@163.com (G.S.); 2 National Higher Education and Research Institute, Hamelmalo Agricultural College, Keren P.O. Box 397, Eritrea; 3 College of Veterinary Medicine, Gansu Agricultural University, Lanzhou 730070, China; 4 Gansu Province Research Center for Basic Disciplines of Pathogen Biology, Lanzhou 730046, China; 5 Key Laboratory of Veterinary Etiological Biology, Key Laboratory of Ruminant Disease Prevention and Control (West), Ministry of Agricultural and Rural Affairs, Lanzhou 730046, China

**Keywords:** Africa, *Brucella*, Brucellosis, meta-analysis, one health, seroprevalence, small ruminants

## Abstract

Brucellosis remains a critical zoonotic threat to public health and livestock economies globally, particularly constraining small ruminant productivity in Africa, yet continental data remain scarce. The objectives of this review are to provide apparent small ruminant brucellosis prevalence/positivity estimates across Africa by country, region, continent, and diagnostic modality. A systematic search was conducted on PubMed, ScienceDirect, and Google Scholar for articles published from 2000 to 2025. Data from 151 eligible studies representing 28 countries were extracted and meta-analyzed. Due to extreme heterogeneity (*I*^2^ > 97%), a single continental pooled estimate is not epidemiologically meaningful. For descriptive reference, Africa’s apparent pooled seroprevalence was 4.9% (95% CI: 3.0–7.5%; prediction interval: 0.1–32.1%), and pooled PCR positivity was 12.7% (95% CI: 6.0–21.9%; prediction interval: 0.8–38.9%). Descriptive regional seroprevalence ranged from 3.3% (95% CI: 1.2–7.4%) (West Africa) to 6.2% (95% CI: 3.8–9.4%) (East Africa). Country-level crude seroprevalence ranged from 0.18% (Morocco) to 19.53% (Libya), with diagnostic method prevalence ranging from 3.37% (2-Mercaptoethanol (2-ME)) to 21.54% (Whole Genome Sequencing (WGS)). Both *B. abortus* and *B. melitensis* are reported with significant variations. Data absence from several African countries highlights a critical knowledge gap. We propose multi-level prevention and control strategies.

## Simple Summary

Brucellosis is a serious zoonotic disease caused by *Brucella* species, affecting both animals and humans globally. It is endemic in African livestock, causing critical economic loss. This systematic review and meta-analysis focused on the prevalence of brucellosis in small ruminants (sheep and goats) across Africa by country, region, continent, and diagnostic methods. Articles published between 2000 and 2025 were systematically searched in PubMed, ScienceDirect, and Google Scholar. All relevant data were collected and meta-analyzed. Due to high heterogeneity (*I*^2^ > 97%), a single pooled estimate for Africa is not epidemiologically valid. Africa’s apparent pooled seroprevalence was 4.9% (95% CI: 3.0–7.5%; prediction interval: 0.1–32.1%), and pooled PCR positivity was 12.7% (95% CI: 6.0–21.9%; prediction interval: 0.8–38.9%). More than half of African countries from all regions reported the presence of small ruminant brucellosis, with significant country-level and regional variations. Both species of *B. abortus* and *B. melitensis* were reported. Knowing the prevalence of small ruminant brucellosis in Africa is crucial for implementing effective prevention and control programs. Therefore, this study calls for a multi-level prevention and control strategies, combining vaccination (mass or targeted regional), tiered WOAH-aligned surveillance, and public awareness aligned with safe animal product handling.

## 1. Introduction

Brucellosis is considered a serious problem in animals and humans, causing a high incidence in Africa [1,2]. It causes significant economic losses across Africa. The economic losses can be direct losses, including late-term abortion, stillbirth, infertility, and animal deaths, and indirect losses, including decreased milk production, animal weight loss, increased veterinary costs (diagnosis and vaccination costs), loss in market value of animals, lost draft power, and losses due to banning the market of infected animals, as well as human suffering resulting from the disease [3,4,5]. Some examples include, in 2024, an individual loss per abortion in small ruminants estimated at United States Dollar (USD) 23.06 (for local species) and USD 70.06 (for non-local species) [6], and in 2025, median annual losses per infected animal of roughly 9.7 international dollars (int. USD) for sheep and 10.6 int. USD for goats [7] were reported in Tanzania. Brucellosis in sheep and goats is mainly caused by *Brucella melitensis*, a Gram-negative facultative intracellular coccobacillus. There are three biovars (1, 2 and 3), and all of these biovars can cause disease in small ruminants [8,9]. The main modes of transmission for *Brucella* species are through ingestion and via the oronasal and conjunctival mucosa; in addition, it can also be transmitted venereally and through broken skin from the primary sources of infection including placenta, fetal fluids, and vaginal discharges expelled by infected ewes and goats during abortion or parturition at full term. *Brucella* species can also shed in udder secretions and semen, and *Brucella* may be isolated from various tissues, such as lymph nodes, heart, spleen, and organs associated with reproduction including uterus, epididymis and testes, as well as from arthritic lesions [9,10]. The main clinical signs of brucellosis in small ruminants are, as in all female ruminants, reproductive failure, abortions (most often during the last trimester), and birth of weak offspring [9,10]. Additionally, other symptoms that may be observed include subclinical mastitis, orchitis, epididymitis, hygroma, arthritis, metritis, and sometimes placental retention [8,9,10].

Brucellosis is generally diagnosed either by direct methods (isolation and identification of *Brucella* spp.) or indirect methods (detection of specific antibodies and/or antigens) [11,12]. The most reliable method and gold standard diagnosis of *Brucella* is the isolation and identification of the bacteria. However, its performance is time-consuming, difficult, and poses a significant infection risk to laboratory workers; thus, to avoid these risks, it necessitates a specific biosafety level three procedures [11,12,13,14]. The diagnosis of Brucella-specific antibodies is conventionally carried out using several serological tests [13]. Serological tests are comparatively faster, easier, and safer; therefore, they have been favorably used for the mass screening of brucellosis infections [15]. They are crucial for the laboratory diagnosis of brucellosis, since most control and eradication programs depend on them [11,12]. Molecular techniques are also important tools for diagnosis and epidemiological studies, providing relevant information for the identification of species and biotypes of *Brucella* spp., which allows for the differentiation between virulent and vaccine strains [12].

Small ruminants (sheep and goats) are vital to Africa’s agricultural economy, providing crucial food security, steady income, and financial resilience for millions of smallholder farmers and rural households. They serve as multipurpose assets, supplying meat, milk, and hides, while acting as easily liquefiable savings accounts in areas with limited banking [16,17]. In 2021, there were over 416 million sheep and over 481 million goats in Africa [18]. Pastoral breeding of small ruminants in Africa refers to extensive, mobile livestock rearing and inbreeding among sheep and goats [18]. Cross-border trade of small ruminants in Africa is vital to pastoralist livelihoods and regional food security [19]. Small ruminants are an integral part of livestock keeping in developing countries, especially in Sub-Saharan Africa, where they are primarily kept for immediate cash sources, milk, meat, wool, manure, and savings or risk distribution. They also serve various social and cultural functions that vary among different cultures, socio-economic conditions, agro-ecologies, and locations in tropical and subtropical Africa [5]. Small ruminants have many advantages over large ruminants for most smallholder farmers, including their prolific and hardy nature, having lower feed costs, quicker turnover, ease of management, and being an appropriate size for slaughter [5,8]. They also exhibit tolerance for less favorable conditions; therefore, they experience far less in mortality during periods of drought compared to large ruminants. Additionally, breeders prefer small ruminants, as the risk of losing large ruminants is too high [20]. In Africa, small ruminants represent cash that can be easily mobilized for current expenditures [8].

Even though small ruminants are important to the livelihoods of producers and national economies in some African countries, the current productivity of small ruminants in developing countries remains low, mainly due to underfeeding, poor management systems, and diseases [5,8]. Brucellosis occupies a higher place among the diseases that constrain small ruminants’ (sheep and goats’) productivity in the world in general and in Africa in particular [5,8]. Brucellosis ranks first on the list of zoonotic bacterial diseases, with more than 500,000 human cases are reported annually worldwide in disease-endemic regions [21]. In many African countries and in Sub-Saharan Africa, little is known about the prevalence of brucellosis, which is not well documented, and data can vary by region, livestock populations, and other factors [22,23]. Understanding the prevalence of brucellosis in small ruminants in African countries, regions, and the entire continent is crucial for providing surveillance data, understanding the role of cross-border transmission of brucellosis across African countries, and recommending the possible necessary prevention and control measures to reduce the impact of the disease on both animal and human health and economic losses.

The objectives of this systematic review and meta-analysis are therefore to provide a transparent, reproducible synthesis of reported apparent prevalence/positivity estimates of brucellosis in small ruminants (sheep and goats) in Africa by country, region, continent, and diagnostic modality.

## 2. Materials and Methods

### 2.1. Literature Search Method

This systematic review and meta-analysis article was prepared in accordance with the Preferred Reporting Items for Systematic Reviews and Meta-analyses (PRISMA) reporting guideline for the design and analysis of the selected and qualified study reports [24]. The protocol for this systematic review and meta-analysis was conducted following the methods described in a prior protocol by Zihan Tian et al. [25]. As our protocol follows that previous protocol, a new registration was not sought. The methods followed are identical to the original protocol, except for the following pre-specified deviations: expanded search dates, differences in animal species, variations in the number of languages, and discrepancies in the number of searching databases. To review the prevalence of small ruminants’ brucellosis in Africa, a literature search was conducted to identify articles published between 2000 and 2025. The aim was to gather a comprehensive collection of articles on the prevalence of small ruminant (sheep and goat) brucellosis in all African countries, regions, and the continent as a whole, published only in English. All articles were retrieved from 1 January to 15 February 2026. Articles were searched online from three databases: PubMed (1–15 January 2026), ScienceDirect (16–30 January 2026), and Google Scholar (31 January–15 February 2026). The articles were searched using the following natural languages of four categories of search terms, including the Boolean operators “AND” and “OR,” where necessary. The utilized search terms were (1) prevalence of small ruminant brucellosis plus the names of all African countries and Africa; (2) prevalence of sheep AND/OR goat brucellosis plus the names of all African countries and Africa; (3) prevalence of caprine AND/OR ovine brucellosis plus the names of all African countries and Africa; and (4) *Brucella melitensis*, *Brucella abortus* AND/OR *Brucella ovis* in sheep AND/OR goats plus the names of all African countries and Africa. Following the initial search formula, we included the following search strategies to filter the articles if they met the following criteria: (1) articles must be original (study) articles, and the study type must be cross-sectional, cohort, or case-control; (2) articles must include at least one search term in their title; and (3) articles must be published within the specified time (2000–2025). Then, all articles were retrieved, manually checked for duplicates, read, and assessed for eligibility. We also excluded grey literature and systematic reviews (meta-analyses) manually.

### 2.2. Articles Inclusion and Exclusion Criteria

The following criteria were established for inclusion in this review article: (1) articles must report small ruminant brucellosis (at least in either sheep or goats) from any African country; (2) reported positive brucellosis must include total number of tested animals plus total number of positive results or positive rates, with the exception of articles that reported the *Brucella* species were included even without the total number of tested animals; (3) articles must mention at least one specific testing method for diagnosis of brucellosis. The articles that only satisfied the specified criteria were selected for further review and analysis. As the reports of brucellosis in African countries vary greatly, this review article included the available reports of brucellosis in small ruminants from the search results, conducted with all the defined diagnostic methods. We categorized the tests into serological, molecular, and *Brucella* culture/isolation methods for eligible qualitative synthesis. In addition, since the reports of *Brucella* species in small ruminants are scarce, all articles reporting *Brucella* species in small ruminants were included without predefined criteria.

### 2.3. Quality Assessment and Data Extraction

Data extracted from each identified study article included the authors, the reporting country, the study year, the appropriate diagnostic methods, the small ruminant species (both sheep and goats), the total sample size, the total number of positive samples, and JBI item scores. The nine questions of the Joanna Briggs Institute (JBI) quality appraisal checklist were as follows. (1) Was the sample frame appropriate to address the target population? (2) Were study participants sampled in an appropriate way? (3) Was the sample size adequate? (4) Were the study subjects and the setting described in detail? (5) Was the data analysis conducted with sufficient coverage of the identified sample? (6) Were valid methods used for the identification of the condition? (7) Was the condition measured in a standard, reliable way for all participants? (8) Was there appropriate statistical analysis? (9) Was the response rate adequate, and if not, was the low response rate managed appropriately? These questions were used to assess the quality of each individual paper among published studies reporting prevalence data [26,27]. Each question was scored as “yes” (1), “no” (0), or “unclear” (0). A total quality score (range 0–9) was calculated for each study. Based on predefined criteria, studies scoring 0–3 were classified as high risk of bias, 4–6 as moderate risk, and 7–9 as low risk of bias. As the included studies scored a minimum of moderate risk of bias, no study was excluded solely based on quality score.

Two authors (W.S.G. and J.G.) independently extracted the data and assessed the quality of the publication. All discrepancies were resolved by one author (G.S.). Then, all the authors (W.S.G.; J.G.; G.S.; F.Z.; and Y.C.) discussed and approved the results together. The analysis was performed in accordance with PRISMA guidelines [24].

### 2.4. Data Analysis

Microsoft Excel was used for the storage of the extracted data. All statistical analyses were then performed using R version 4.3.2 with the meta package (version 6.5.0). Forest plots were generated using the forest function within the same package. Due to anticipated clinical and statistical heterogeneity across the included countries, a random-effects meta-analysis model was employed. The pooled prevalence of positive tests was calculated using the Freeman–Tukey double arcsine transformation to stabilize variances, with inverse variance weighting. Given recent methodological debate on transformation-based meta-analysis of proportions [28,29], we assessed whether the Freeman–Tukey approach influenced the pooled estimates and confidence intervals by comparing it with two alternative methods: logit transformation and a binomial-normal generalized linear mixed model (GLMM) without transformation. All three methods yielded similar results, confirming the robustness of the findings (see Section 3). The primary analysis used the DerSimonian-Laird (DL) estimator for the between-study variance. Individual study 95% confidence intervals were calculated using the Wilson score method, and the overall pooled effect is reported with its 95% confidence interval (CI).

Heterogeneity was assessed using the *I*^2^ statistic and Cochran’s Q test (*p* < 0.10 considered significant). Given the substantial heterogeneity observed (*I*^2^ = 99.5%), we assessed the robustness of the pooled estimates by repeating the random-effects meta-analysis using two alternative between-study variance estimators: Restricted Maximum Likelihood (REML) and Paule–Mandel (PM), each with the Hartung–Knapp–Sidik–Jonkman (HKSJ) adjustment for confidence intervals and *p*-values. Material changes in pooled estimates, confidence intervals, or conclusions across estimators were evaluated and are reported in the Section 3. The high heterogeneity was further explored through regional subgroup analysis and meta-regression analysis. Given the limited number of studies available for certain subgroups (e.g., Southern Africa: *n* = 3; Central Africa: *n* = 1) and the low statistical power of meta-regression with small sample sizes, only country-level descriptive results are presented. The meta-analysis was conducted in accordance with the PRISMA guidelines [24,30].

### 2.5. Publication Bias and Sensitivity Analysis

Publication bias was assessed visually using funnel plots for each region with at least three studies (East Africa, North Africa, West Africa, and Sub-Saharan Africa). Egger’s regression test was performed only for Sub-Saharan Africa (22 studies), as this test is unreliable with fewer than 10 studies [31]. For Sub-Saharan Africa, the funnel plot appeared roughly symmetrical, and Egger’s test was not significant (*z* = 1.52, *p* = 0.128), suggesting no strong evidence of small-study effects. For East Africa (*n* = 9 studies), North Africa (*n* = 6 studies), and West Africa (*n* = 8 studies), the funnel plots were inspected descriptively, but Egger’s test was not performed due to the very limited number of studies, which would render any *p*-values statistically unreliable. Across all regions, there was no visual evidence of gross asymmetry, although the small number of studies in most regions precludes definitive conclusions about publication bias.

A series of sensitivity analyses for the pooled seroprevalence were conducted at the country level (each country contributed a combined serological study estimate). Leave-one-out analyses showed that no single country disproportionately influenced the pooled prevalence for any region, with all excluded estimates remaining within one percentage point of the original pooled estimate (see Supplementary Table S1 for complete leave-one-out results). Influence diagnostics (Cook’s distance, DFFITS) identified no overly influential studies. A comparison of fixed-effect versus random-effects models revealed substantial differences in North Africa (fixed-effect: 7.2% vs. random-effects: 4.9%), confirming that the random-effects model is more appropriate given the high heterogeneity. Outlier detection identified Kenya (13.8%) in East Africa and Libya (19.5%) in North Africa as potential outliers; however, their exclusion did not materially change the regional pooled estimates (East Africa without Kenya: 5.4% vs. 6.2%; North Africa without Libya: 3.6% vs. 4.9%).

## 3. Results

### 3.1. Literature Search and Results of Included Articles

**Figure 1:**
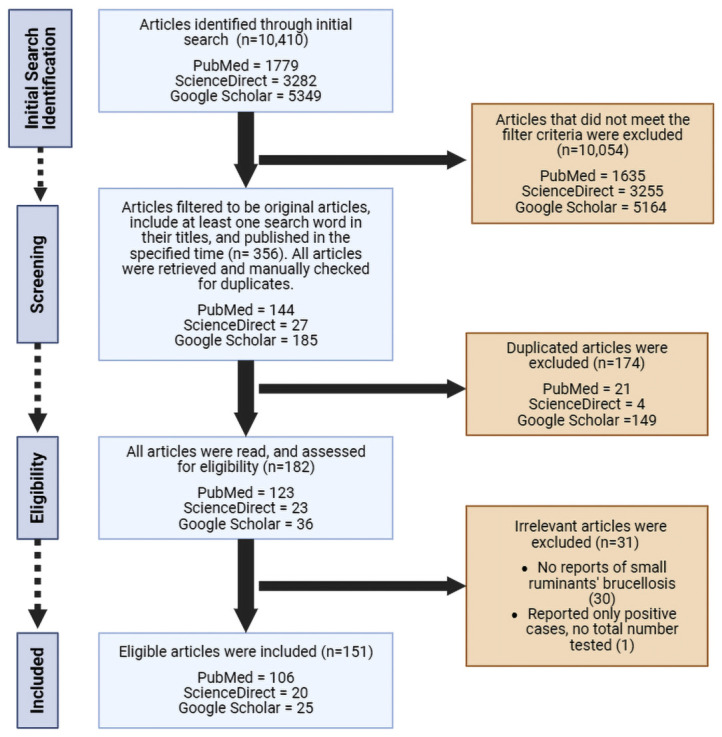
Flow diagram of literature search, screening, and article selection methods for small ruminants’ brucellosis in Africa.

The initial database search using the search terms described above (2.1) for this review study identified a total of 10,410 articles that related to general brucellosis in all African countries and generally pertained to African countries. Following the initial search, articles were filtered using the filter criteria described in Section 2.1, and a total of 10,054 studies were excluded for not fulfilling the filtering criteria, with 356 articles from all African countries that met the filtering criteria being retrieved. Duplicate articles (duplicate records identified in two or more databases, *n* = 174) were then excluded, resulting in a total of 182 articles further retrieved. Based on the predefined inclusion criteria (2.2) and quality assessment of the retrieved articles, a total of 31 studies (30 for no report of small ruminant brucellosis and 1 for reporting only positive cases, no total number tested) were excluded, resulting in 151 qualified articles being selected for inclusion in this systematic review and meta-analysis study (Figure 1). From the total 151 included articles, 128 were classified as being at low risk of bias, and 23 were classified as being at moderate risk of bias.

### 3.2. Prevalence of Brucellosis in Small Ruminants in African Countries

#### 3.2.1. Ethiopia

Small ruminant brucellosis is widespread across all regions of Ethiopia, hampering their productivity [32]. Many cross-sectional serological studies have been conducted in various locations in Ethiopia and at different times to investigate the seroprevalence of brucellosis in small ruminants. Most of these studies utilized the Rose Bengal Plate Test (RBPT) as a screening test and Complement Fixation Test (CFT) as a second-tier test, considering it a confirmatory test, with a few of them using the enzyme-linked immunosorbent assay (ELISA) as a second-tier test for the RBPT screening test. A total of 46 studies reported the prevalence of brucellosis in small ruminants from different locations in Ethiopia, employing either one or a combination of the diagnostic tests, including RBPT, CFT, ELISA, and polymerase chain reaction (PCR) tests (Table 1). In addition, both the species *Brucella abortus* and *B. melitensis* were identified and reported in Ethiopia, with a total of 43 isolates characterized by whole-genome sequencing (WGS) as *B. melitensis* [33,34], and 72/704 (10.18%) were positive for *B. abortus* and *B. melitensis* using PCR [33,35,36]. Therefore, the crude total prevalence of brucellosis in small ruminants in Ethiopia is estimated at 5.22% (4336/83,080) using all three serological tests (RBPT, CFT, and ELISA), and the PCR positivity rate is estimated at 11.44% (107/935).

#### 3.2.2. Nigeria

In Nigeria, several serological investigations have shown that *Brucella* infection is endemic in the livestock population [78]. A total of 17 studies conducted in different locations in Nigeria and at various times to investigate brucellosis reported the prevalence of brucellosis in small ruminants using different diagnostic methods. Most of these studies used RBPT as a screening test and ELISA as a second-tier test. All of these studies reported the prevalence of brucellosis in small ruminants using RBPT, ELISA, STAT, and PCR (Table 1). In addition, a total of 165/1320 (12.50%), 14/415 (3.37%), and 14/108 (12.96%) brucellosis-positive small ruminants were reported using a Lateral Flow Assay (LFA), 2-Mercaptoethanol (2-ME), and Milk Ring Test (MRT), respectively [77,81,87,91,92]. The *Brucella* species of *B. abortus* was also identified and reported using both *Brucella* Cultural Isolation (BCI) method with 5/28 (17.86%) positive [86], and PCR method with 5/67 (7.46%) positive [88,89]. Therefore, the total brucellosis prevalence in small ruminants in Nigeria is 10.02% (2061/20,579) using the combination of the tests RBPT, ELISA, STAT, LFA, and 2-ME, with estimated PCR positivity rates of 10.92% (13/119).

#### 3.2.3. Egypt

Brucellosis is an endemic disease in both livestock and humans in Egypt for thousands of years [97]. A total of 16 studies (eligible for this review study) conducted in Egypt at various times reported the prevalence of small ruminant brucellosis using the most commonly used diagnostic tests, including RBPT, CFT, ELISA, PCR, and STAT (Table 1). Other small ruminant brucellosis reports in Egypt used the Buffered Acidified Plate Antigen Test (BAPAT) 105/1789 (5.87%) [97,131] and the MRT 41/610 (6.72%) [97]. Both the species of *B. abortus* and *B. melitensis* were identified and reported in Egypt using the Bacterial Strain Isolation (BSI) method 34/144 (23.61%) [131,132], the WGS method 3/3 (100%) [101], and the PCR technique 53/53 (100%) [100,101,107]. The overall small ruminant brucellosis rate in Egypt is therefore 2.32% (1437/61,874) using a combination of the RBPT, CFT, ELISA, STAT, and BAPAT test, with PCR positivity rates of 100% (53/53).

#### 3.2.4. Uganda

Brucellosis is endemic in Uganda, impacting the health of humans and animals and ultimately affecting the national economy [111]. A total of nine studies from Uganda reported the seroprevalence of brucellosis in small ruminants using the most common serological tests, including RBPT, ELISA, and STAT (Table 1). The crude total small ruminants’ brucellosis seroprevalence in Uganda is therefore 6.97% (489/7013) using a combination of RBPT, ELISA, and STAT tests. A total of 51/925 (5.51%) of small ruminants with brucellosis were also reported using the Native Hapten immunoprecipitation test (NHIP) [116].

#### 3.2.5. Tanzania

Brucellosis is endemic in Tanzania, with studies indicating a significant production losses and potential public health problems [133]. A total of eight studies conducted in Tanzania at different times reported the prevalence of brucellosis in small ruminants in Tanzania using the most commonly employed serological tests, including RBPT and ELISA, as well as PCR techniques (Table 1). The overall brucellosis prevalence in small ruminants in Tanzania is therefore estimated at 3.26% (692/21,231) using both RBPT and ELISA tests. A total of 3/3 (100%) cases of *B. melitensis* were also identified in Tanzania using the PCR method [122].

#### 3.2.6. Kenya

Brucellosis is endemic in Kenya and causes major issues affecting both livestock production and humans [129,130]. A total of seven studies from Kenya reported the prevalence of small ruminant brucellosis at different times and different places in Kenya using RBPT, ELISA, and PCR methods (Table 1). The overall prevalence of small ruminant brucellosis in Kenya is estimated at 13.79% (722/5237) using both RBPT and ELISA tests, with PCR positivity rates of 21.28% (136/639). Both species *B. abortus* and *B. melitensis* were also identified and reported using the PCR technique in Kenya [126,128].

#### 3.2.7. Algeria

In Algeria, small ruminant brucellosis remains a persistent enzootic threat despite the extensive *Brucella* control efforts conducted in the country, including a large vaccination campaign from 2006 to 2016 [134]. A total of five articles qualified for this review from Algeria reported the prevalence of small ruminant brucellosis using different methods and at different times. The overall prevalence of brucellosis in small ruminants in Algeria was 1.04% (53/5105), 13.46% (7678/57,042), and 54.74% (52/95) using RBPT, CFT, and ELISA tests, respectively [134,135,136,137,138]. Therefore, the crude total brucellosis prevalence in small ruminants in Algeria is estimated at 12.50% (7783/62,242) using RBPT, CFT, and ELISA tests. The species *B. melitensis* was also identified using the whole-genome sequencing (WGS) method, with a total of 36/90 (40.00%) [134,138].

#### 3.2.8. Sudan

Brucellosis in Sudan occurs in all farm animals, some wildlife animal species, and in humans [139]. A total of five study articles that qualified for this review study reported brucellosis in small ruminants. The seroprevalence of small ruminant brucellosis in Sudan is 3.32% (119/3588) and 3.31% (30/907) using RBPT and ELISA tests, respectively [139,140,141,142,143]. The pooled seroprevalence of brucellosis in small ruminants in Sudan is then estimated to be 3.31% (149/4495) using both RBPT and ELISA serological tests.

#### 3.2.9. Libya

Brucellosis is considered to be endemic in animals including small ruminants, cattle, and camels in Libya [144,145]. Four articles that qualified for this review study reported positive results for small ruminants. The overall seroprevalence of small ruminant brucellosis was 28.22% (149/528) and 17.58% (413/2349) using RBPT and ELISA, respectively, which resulted in a pooled seroprevalence of 19.53% (562/2877) in Libya [144,145,146,147].

#### 3.2.10. South Africa

Brucellosis in South Africa is a reported disease in animals (cattle, sheep, and goats) and in humans, with both *B. abortus* and *B. melitensis* identified [148]. A total of four studies reported small ruminant brucellosis, resulting in an overall brucellosis rate in South Africa of 1.46% (1018/69,639) and 14.50% (29/200) using CFT and PCR techniques (the PCR technique also identified both *B. abortus* and *B. melitensis* species in South Africa) [148,149]. In addition to the above reports, rates of 5.32% (119/2238) of both *B. abortus* and *B. melitensis* species were also recorded in South Africa using the BSI method [150,151].

#### 3.2.11. Tunisia

Brucellosis is endemic and poses public health threats in Tunisia despite control measures such as ongoing surveillance and vaccination programs being conducted [152]. Of the total of four qualified articles that reported small ruminant brucellosis in Tunisia, the prevalence of small ruminant brucellosis was 14.63% (24/164), 14.84% (142/957), and 11.74% (100/852) using RBPT, ELISA, and PCR methods, respectively [153,154,155]. The overall prevalence of small ruminant brucellosis in Tunisia is estimated at 14.81% (166/1121). Regarding *Brucella* species, both *B. abortus* and *B. melitensis* at a rate of 100/852 (11.74%) were isolated using PCR techniques [153,154], and 2/2 (100%) were isolated using the WGS method [152].

#### 3.2.12. Eritrea

In Eritrea, brucellosis is endemic, as proven by several seroprevalence studies in both domestic animals and humans [156]. Of the three articles suitable for this study, the prevalence of small ruminant brucellosis in Eritrea was 6.98% (80/1146), 2.75% (21/765), 12.58% (39/310), and 3.51% (8/228) using RBPT, CFT, ELISA, and PCR methods, respectively [156,157,158]. The total prevalence of small ruminant brucellosis in Eritrea is therefore estimated to be 6.30% (140/2221) using the combination of the tests RBPT, CFT, ELISA, with a PCR positivity rate of 3.51% (8/228). The PCR technique also identified both *B. abortus* and *B. melitensis* species in Eritrea [156,157].

#### 3.2.13. Rwanda

Brucellosis is a significant yet underreported disease in Rwanda [159]. A total of three qualified study articles were included in this study, and they all reported the presence of small ruminant brucellosis in Rwanda. The prevalence of brucellosis in small ruminants in Rwanda was 8.36% (52/622), 11.15% (102/915), and 100% (4/4) using RBPT, ELISA, and PCR methods, respectively [159,160,161]. The overall prevalence of small ruminant brucellosis in Rwanda is estimated at 10.02% (154/1537) using both RBPT and ELISA tests, with a PCR positivity rate of 100% (4/4). The PCR technique also recognized both *B. abortus* and *B. melitensis* species in Rwanda [161].

#### 3.2.14. Cameroon

Two articles qualified for this review reported the presence of small ruminant brucellosis in Cameroon. Brucellosis prevalence in small ruminants in Cameroon is 2.20% (119/5409), 4.24% (35/825), and 0.76% (35/4584) using RBPT, ELISA, and PCR techniques, respectively [162,163]. The overall prevalence of small ruminant brucellosis in Cameroon is therefore estimated at 2.47% (154/6234) using both the RBPT and ELISA tests, with a PCR positivity rate of 0.76% (35/4584). The PCR method also identified the species *B. abortus* in Cameroon [163].

#### 3.2.15. Côte D’Ivoire

Two study articles reported small ruminant brucellosis in Côte d’Ivoire using three different serological tests. The seroprevalence of brucellosis in small ruminants in Côte d’Ivoire is 5.45% (44/807), 0.80% (5/622), and 11.70% (22/188) using RBPT, ELISA, and Fluorescence Polarization Assay (FPA), respectively, resulting in an overall prevalence of 4.39% (71/1617) of small ruminant brucellosis in Côte d’Ivoire [164,165].

#### 3.2.16. Mali

Two relevant studies for this review reported the presence of brucellosis in small ruminants from Mali. The serological prevalence of brucellosis in small ruminants in Mali is 0.33% (2/608) and 4.07% (35/860) using RBPT and ELISA, respectively [166,167], resulting in an overall prevalence of small ruminants’ brucellosis of 2.52% (37/1468).

#### 3.2.17. Namibia

Two articles that reported brucellosis in small ruminants from Namibia were qualified for this study. Brucellosis prevalence in small ruminants in Namibia is 0.14% (32/22,994) and 0.29% (70/24,547) using RBPT and CFT, respectively, which resulted in the overall prevalence of brucellosis in Namibia being 0.21% (102/47,541) [168,169].

#### 3.2.18. Somalia

Brucellosis is a neglected disease and is a disease that impedes small ruminant production in Somalia. From the two qualified articles reporting small ruminant brucellosis in Somalia, the prevalence of small ruminant brucellosis is 7.20% (57/792) and 3.03% (24/792) using RBPT and ELISA, respectively [170,171]. The total seroprevalence of brucellosis in small ruminants in Somalia is therefore estimated to be 5.11% (81/1584).

#### 3.2.19. South Sudan

Brucellosis is a neglected disease reported in both animals and humans that has serious public health implications and may incur significant economic losses in pastoral rural areas of South Sudan [172,173]. The prevalence of small ruminant brucellosis in South Sudan is 9.04% (33/365) using both RBPT and ELISA tests, and 3.03% (1/33) using the PCR technique [172,173]. The overall prevalence is therefore estimated at 9.04% (66/730) using both RBPT and ELISA tests, and the PCR positivity rate is 3.03% (1/33). The PCR method also identified *B. abortus* species from one goat in South Sudan [173].

#### 3.2.20. Zambia

Brucellosis is an endemic disease in Zambia that is reported in both animals and humans [174]. Two qualified study articles from Zambia reported the total prevalence of small ruminant brucellosis in Zambia to be 9.20% (110/1196) using ELISA test [174,175].

#### 3.2.21. Botswana

Small ruminants’ brucellosis was reported in Botswana with a total prevalence of 18.25% (23/126) using PCR techniques, with an animal species prevalence of 17.85% (20/112) in goats and 21.41% (3/14) in sheep [176].

#### 3.2.22. Burkina Faso

From the total of 600 (300 sheep and 300 goats) serum samples tested in Burkina Faso, the prevalence of small ruminants’ brucellosis was 2.00% (12/600) and 5.16% (31/600) using RBT and ELISA, respectively [177]. The total brucellosis seroprevalence in Burkina Faso is therefore 3.58% (43/1200) using both tests utilized in Burkina Faso [177].

#### 3.2.23. Djibouti

Brucellosis is a formidable obstacle to livestock production and public health in Djibouti. A total seroprevalence of 3.41% (6/176) of small ruminant brucellosis from goat samples was reported using RBPT in Djibouti [178].

#### 3.2.24. Gambia

From the total of 1123 small ruminants (744 goats and 379 sheep) tested in Gambia, a total seroprevalence of 1.25% (14/1123) was found using RBPT [179].

#### 3.2.25. Ghana

A total prevalence of small ruminant brucellosis of 13.24% (49/370) and 16.61% (53/319) was reported from Ghana using RBPT and modified Ziehl–Neelsen staining (MZN) methods [180].

#### 3.2.26. Guinea

From the total of 894 small ruminants (408 goats and 486 sheep) tested in Guinea, a total seroprevalence of 0.22% (2/894) was found using the ELISA test [181].

#### 3.2.27. Morocco

Brucellosis in small ruminants is an endemic disease in Morocco. From a total of 1095 small ruminants (51 goats and 1044 sheep) tested in Morocco, a total seroprevalence of 0.18% (2/1095) was found using modified RBPT [182].

#### 3.2.28. Niger

**Figure 2:**
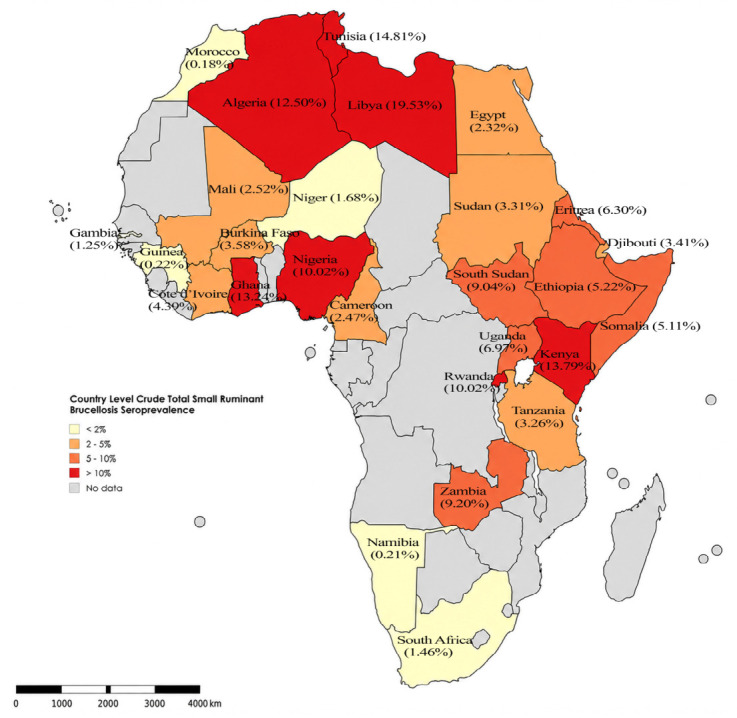
Map of country-level crude total small ruminant brucellosis seroprevalence in Africa. Data source: systematic search of PubMed, ScienceDirect, and Google Scholar (2000–2025). The 27 included countries and their corresponding 150 cited studies are listed in “Section 3. Results, Section 3.2. Prevalence of Brucellosis in Small Ruminants in African Countries” of this article. The map was created using MapChart (https://www.mapchart.net/africa.html, accessed on 4 June 2026). Administrative boundary data are sourced from the MapChart internal database, optimized with Inkscape (Version 1.4.4). Map projection: Miller.

A total of 2025 small ruminant sera (839 goats and 1186 sheep) were examined in Niger, resulting in a total prevalence of 1.68% (34/2025) of small ruminant brucellosis using the ELISA test in Niger [183]. Total crude small ruminant brucellosis seroprevalence for all reporting countries is presented in Figure 2.

### 3.3. The Entire Africa’s Prevalence of Small Ruminants’ Brucellosis

Small ruminants’ brucellosis is reported in more than half (28) of the African countries using 15 different brucellosis diagnostic tests. We stratified the results into three groups, including serological, molecular (PCR), and bacteriological identification test results. Then, we analyzed the pooled prevalence for the combined serological tests and pooled positivity rates of the PCR results. However, the bacteriological isolation test results are only reported numerically, without meta-analysis. A total of 27 African countries reported a total of 410,399 small ruminant samples tested, of which 20,480 were positive for brucellosis using the combination of serological tests, including RBPT, CFT, ELISA, STAT, BAPAT, LFA, 2-ME, and FPA. The observed country level small ruminant brucellosis seroprevalence ranged from 0.18% (Morocco) to 19.53% (Libya) (Section 3.2). Extreme heterogeneity was present across countries (*I*^2^ = 99.5%, *p* < 0.001), indicating that the observed variation reflects true differences in prevalence, animal populations, sampling strategies, and study designs rather than random error. Therefore, a single continental pooled prevalence is not epidemiologically meaningful as a representative value for Africa. For descriptive purposes only (e.g., comparison with other meta-analyses), a random-effects meta-analysis using the Freeman–Tukey double arcsine transformation yielded a pooled small ruminant brucellosis prevalence of 4.9% (95% CI: 3.0–7.5%) in Africa, using the combination of the listed serological tests. However, due to the very high heterogeneity, this pooled estimate should be considered descriptive only and interpreted with caution. The 95% prediction interval (0.1–32.1%) indicates that the true prevalence in a future setting could vary widely, so the pooled mean is not a reliable predictor. Therefore, we do not report the serological pooled estimate as a primary finding but only as a descriptive summary for cross-continental comparison.

To assess whether the Freeman–Tukey double arcsine transformation influenced the pooled estimates and confidence intervals, we compared it with two alternative methods: logit transformation and a binomial-normal generalized linear mixed model (GLMM) without transformation. The pooled prevalence estimates were as follows: Freeman–Tukey = 4.93% (95% CI: 3.03–7.46%), logit = 5.01% (95% CI: 2.90–7.79%), and GLMM = 4.92% (95% CI: 3.12–7.42%). All three methods yielded overlapping confidence intervals and consistent conclusions, confirming that the choice of transformation did not materially influence the findings (Supplementary Table S2).

To assess whether the choice of between-study variance estimator influenced this result despite the extreme heterogeneity, we compared the primary DerSimonian–Laird (DL) estimator with REML + HKSJ and Paule–Mandel + HKSJ estimators. The pooled prevalence estimates were virtually identical across all three methods: DL = 4.9% (95% CI: 3.0–7.5%), REML + HKSJ = 5.1% (95% CI: 2.8–8.1%), and Paule–Mandel + HKSJ = 5.0% (95% CI: 2.9–7.9%). The 95% confidence intervals overlapped substantially, and no material change in statistical conclusion or clinical interpretation occurred (Supplementary Table S3). Thus, the primary DL-based estimate is robust to alternative estimators despite the high heterogeneity.

A total of 12 countries reporting PCR results for small ruminant brucellosis comprising 7893 small ruminant samples were included in the meta-analysis of PCR positivity rates. PCR positivity rates ranged from 0.8% (Cameroon) to 100% (Egypt and Rwanda). The 100% results came from very small, targeted samples (Egypt, *n* = 53; Rwanda, *n* = 4) that represent high-risk foci rather than population prevalence. When these two non-representative studies are excluded, the range among the remaining 10 countries was 0.8–21.3%, with a median of 7.5%. Given the extreme heterogeneity (*I*^2^ = 97.8% including all 12 countries; *I*^2^ = 96.4% after excluding outliers), no single pooled PCR prevalence is epidemiologically meaningful. Therefore, we do not report a pooled estimate as a primary finding. For descriptive purposes only (e.g., comparison with other meta-analyses), a random-effects pooled estimate including all 12 countries was 12.7% (95% CI: 6.0–21.9%; prediction interval: 0.8–38.9%), and after excluding outliers, it was 9.1% (95% CI: 5.2–14.1%). However, due to persistent severe heterogeneity, these estimates are presented only as descriptive summaries of the included data, not as reliable predictors of true prevalence in any specific country or future setting, and should be interpreted with caution. The wide variation across countries may reflect differences in country-level prevalence, sampling strategies, epidemic timing, population risk, and testing protocols.

Two of the most common *Brucella* species that cause brucellosis in small ruminants, namely, *B. melitensis* and *B. abortus*, were recognized and reported using four diagnostic methods—WGS, BSI, BCI, and PCR techniques—which ascertain that both of the species are circulating throughout Africa. In addition to the above results, small ruminant brucellosis is also reported using MZN, MRT, and NHIP in different African countries (Table 2). The total tests utilized in small ruminant brucellosis diagnosis along with the total number of samples tested and their results are presented in Table 2. The total number may include double counting of animals due to possible duplicate test results.

### 3.4. Regional Prevalence of Small Ruminants’ Brucellosis in Africa

As the small ruminant pooled prevalence rate and pooled PCR positivity rate for the whole of Africa produced high heterogeneity, and to estimate the regional prevalence of brucellosis in small ruminants in Africa, all the reporting countries were grouped into their respective regions, and the positive results were totaled for subgroup regional analysis.

#### 3.4.1. East Africa

East Africa comprised nine countries, with a total of 122,809 small ruminants tested, of which 6686 (5.44%) were positive. Observed country-level small ruminant brucellosis seroprevalences ranged from 3.26% (Tanzania) to 13.79% (Kenya). Extreme heterogeneity was present across countries (*I*^2^ = 97.8%, 95% CI: 96.9–98.5%; *Q* = 361.4, df = 8, *p* < 0.001; *τ*^2^ = 0.098). Using a random effects model with the Freeman–Tukey double arcsine transformation, the pooled small ruminant brucellosis seroprevalence was 6.2% (95% CI: 3.8–9.4%), utilizing a combination of serological tests, including RBPT, CFT, ELISA, and STAT. However, due to the very high heterogeneity, this pooled estimate should be considered descriptive only and interpreted with caution. The 95% prediction interval (1.3–19.8%) indicates that a future study in an East African country could yield a prevalence of small ruminant brucellosis as low as 1.3% or as high as 19.8%. This wide prediction interval, together with the high *I*^2^, suggests that the true small ruminant brucellosis seroprevalence varies substantially across East African nations, likely due to differences in country-level prevalence, sampling methods, diagnostic criteria, risk population characteristics, or years of data collection.

For the PCR positivity rate, six countries from East Africa comprising 1959 small ruminant samples were analyzed. The small ruminant brucellosis pooled PCR positivity prevalence in East Africa was 19.7% (95% CI: 5.2–41.5%), with very high heterogeneity (*I*^2^ = 97.6%, *p* < 0.001). The prediction interval ranged from 0.2% to 78.6%. Rwanda reported 100% positivity (4/4), representing targeted sampling of high-risk small ruminants. After excluding Rwanda for a sensitivity analysis, the small ruminant brucellosis pooled estimate PCR positivity rate for East Africa was 12.5% (95% CI: 6.1–20.9%) (*I*^2^ = 94.1%). Individual country rates varied, as follows: Kenya 21.3% (136/639), Tanzania 20.8% (25/120), Ethiopia 11.4% (107/935), Eritrea 3.5% (8/228), and South Sudan 3.0% (1/33).

Both *B. abortus* and *B. melitensis* species were reported from East African countries using WGS and PCR techniques, which indicates that both of them are circulating in the East African region. Additionally, small ruminant brucellosis is also reported using NHIP in Uganda.

#### 3.4.2. North Africa

North Africa included six countries (Algeria, Libya, Egypt, Tunisia, Sudan, Morocco) with a total sample of 133,704 small ruminants and 10,099 positive cases (raw average 7.55%). Observed small ruminant brucellosis prevalences ranged from 0.18% (Morocco) to 19.53% (Libya). Extreme heterogeneity was detected (*I*^2^ = 99.6%, 95% CI: 99.3–99.8%; *Q* = 1247.8, df = 5, *p* < 0.001; *τ*^2^ = 0.187). The random-effects small ruminant brucellosis pooled seroprevalence was 4.90% (95% CI: 1.8–10.7%), using a combination of serological tests, namely RBPT, CFT, ELISA, STAT, and BAPAT. However, due to the very high heterogeneity, this pooled estimate should be considered descriptive only and interpreted with caution. The 95% prediction interval was extremely wide (0.2–38.9%), indicating that the true prevalence in a future North African setting is highly uncertain.

Regarding the PCR results, two countries from North Africa were reported. Pooling was not performed due to extreme heterogeneity and clear sampling differences, and results were presented separately. Egypt reported 100% PCR positivity (53/53). Tunisia reported 11.7% positivity (100/852; 95% CI: 9.6–14.1%), which likely represents a more general population estimate for the region.

Both the species of *B. abortus* and *B. melitensis* were identified and reported using BSI, WGS, and PCR methods, proving that both are circulating in the North African region. Additionally, small ruminant brucellosis is also reported using MRT in Egypt.

#### 3.4.3. West Africa

West Africa comprised eight countries (Nigeria, Burkina Faso, Niger, Ghana, Mali, Gambia, Côte d’Ivoire, Guinea) with 29,276 small ruminant samples tested and 2311 positive cases (raw average 7.89%). Observed small ruminant brucellosis prevalences ranged from 0.22% (Guinea) to 13.24% (Ghana). Extreme heterogeneity was present (*I*^2^ = 98.4%, 95% CI: 97.5–99.0%; *Q* = 438.9, df = 7, *p* < 0.001; *τ*^2^ = 0.142). The random-effects small ruminant brucellosis pooled seroprevalence was 3.3% (95% CI: 1.2–7.4%), using a combination of serological tests, including RBPT, ELISA, STAT, LFA, 2-ME, and FPA. However, due to the very high heterogeneity, this pooled estimate should be considered descriptive only and interpreted with caution. The 95% prediction interval (0.1–30.3%) suggests that a future West African study could find a prevalence anywhere from near zero to nearly one-third of the population tested. Ghana and Nigeria (13.2% and 10.0%, respectively) are notable outliers on the high end, while Guinea and Gambia (0.2% and 1.3%) are outliers on the low end.

A single country, Nigeria (*n* = 119) reported the small ruminant brucellosis PCR positivity rate of 10.9% (95% CI: 6.0–17.5%) (13/119). No pooling was performed.

Both *B. abortus* and *B. melitensis* species were identified and reported from the West African region using BCI and PCR methods, indicating that both the species are circulating in the region. In addition to the above results, small ruminant brucellosis is also reported using MRT (Nigeria) and MZN (Ghana).

#### 3.4.4. Southern Africa

In Southern Africa, three countries (South Africa, Namibia, Zambia) contributed a total of 118,376 small ruminant samples tested, with 1230 positive cases. The observed small ruminant brucellosis prevalences varied substantially, at 0.21% (Namibia), 1.04% (South Africa), and 9.20% (Zambia) as a raw average across the three countries. Due to the small number of countries (only 3), a random-effects meta-analysis model would be unreliable for estimating a pooled prevalence. Therefore, only country-level descriptive results are presented. Extreme heterogeneity was noted, at *I*^2^ = 99.5%, 95% CI: 98.8–99.8%; *Q* = 370.5, df = 2, *p* < 0.001; *τ*^2^ = 0.172, though this statistic is also unstable with only three studies. These findings highlight the need for additional prevalence data from other countries in the region before a quantitative synthesis is justified.

In Southern Africa, two countries (South Africa and Botswana) contributed a total of 326 small ruminant samples tested by PCR. Country-specific PCR positivity prevalences were 14.5% (29/200) for South Africa and 18.3% (23/126) for Botswana. A meta-analytic pooled estimate was not calculated because only two countries were available, which is insufficient for a reliable random-effects model. Descriptive country-level data are therefore presented as the primary findings.

Both *B. abortus* and *B. melitensis* species were also identified and reported from the Southern African region using the BSI method, which ascertains that both of the species are circulating in the Southern African region.

#### 3.4.5. Central Africa

Regional estimates are not possible. Only one country from Central Africa (Cameroon) contributed data. Therefore, no meta-analysis was performed, and no regional pooled prevalence can be reported. The following results pertain solely to Cameroon. Cameroon tested 6234 small ruminant samples (154 positive cases) using RBPT and ELISA. The observed brucellosis seroprevalence was 2.47% (exact binomial 95% CI: 2.11–2.89%).

A separate PCR study in Cameroon (*n* = 4584) reported very low *Brucella* DNA positivity of 0.8% (95% CI: 0.5–1.1%; 35/4584). These findings apply only to Cameroon and cannot be generalized to Central Africa as a region. Additional studies from other Central African countries are needed before any regional estimate can be considered.

Only *B. abortus* was recognized and reported from Cameroon using the PCR technique.

#### 3.4.6. Sub-Saharan Africa

Sub-Saharan Africa (SSA) included 22 countries, with 281,190 small ruminants and 10,530 positives (observed raw proportion 3.74%). The observed country-level prevalences ranged from 0.21% (Namibia) to 13.79% (Kenya). Extreme heterogeneity was present (*I*^2^ = 98.9%, 95% CI: 98.5–99.2%; *Q* = 1930.6, df = 21, *p* < 0.001; *τ*^2^ = 0.121). A random-effects model gave a pooled seroprevalence of 3.60% (95% CI: 2.2–5.7%), using a combination of serological tests, including RBPT, CFT, ELISA, STAT, LFA, 2-ME, and FPA. However, due to the very high heterogeneity, this pooled estimate should be considered descriptive only and interpreted with caution. The 95% prediction interval (0.2–27.8%) is the more informative result, and the true prevalence in a future SSA study could plausibly range from as low as 0.2% to as high as 27.8%. Given this enormous uncertainty, the pooled point estimate should not be interpreted as a stable or precise regional summary. Therefore, we emphasize the descriptive country-level range (0.21–13.79%) rather than the pooled estimate. No ranking of SSA against other regions is attempted, as the prediction intervals overlap substantially with those of other subregions.

Regarding the PCR results, ten countries comprising 6988 small ruminant samples were included. The pooled PCR positivity rate was 9.70% (95% CI: 4.0–17.7%), with substantial heterogeneity (*I*^2^ = 98.3%, *p* < 0.001). However, due to the very high heterogeneity, this pooled estimate should be considered descriptive only and interpreted with caution. The 95% prediction interval (0.7–32.4%) indicates that a future SSA PCR study could yield a prevalence anywhere from <1% to nearly one-third. After excluding the small targeted sampling study from Rwanda (100%, *n* = 4) as a sensitivity analysis, the pooled estimate changed to 7.70% (95% CI: 3.1–13.9%), but the prediction interval remained extremely wide, and heterogeneity persisted (*I*^2^ = 98.0%). These wide prediction intervals underscore that no single pooled estimate can meaningfully summarize SSA. The variation across countries likely reflects genuine differences in epidemiology, sampling methods, laboratory protocols, and timing relative to outbreaks. We, therefore, avoid ranking SSA against other regions or claiming precision for these pooled estimates.

In the Sub-Saharan African region, both *B. abortus* and *B. melitensis* species were identified using four diagnostic methods—WGS, BSI, BCI, and PCR—which ascertained that both of the species are prevalent in the region. In addition to the above results, small ruminant brucellosis is also reported using NHIP (Uganda), MRT (Nigeria), and MZN (Ghana) (Table 3). The total tests utilized, the total number of samples tested and their results for the Sub-Saharan region are described in Table 3.

**Figure 3:**
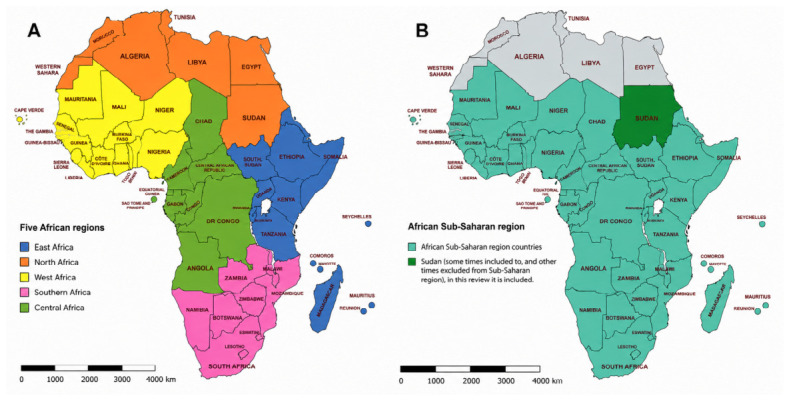
Map of African regions: ((**A**) five African regions; (**B**) African Sub-Saharan region). This map was created using MapChart (https://www.mapchart.net/africa.html, accessed on 4 June 2026). Administrative boundary data are sourced from the MapChart internal database, optimized with Inkscape. Map projection: Miller.

**Figure 4:**
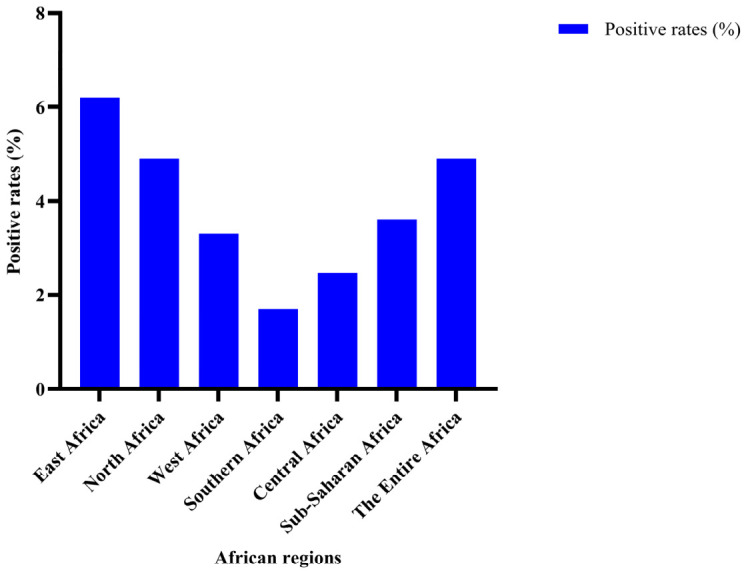
Comparison of the pooled seroprevalence of brucellosis in small ruminants across five African regions, the Sub-Saharan region, and the whole of Africa.

The map of the five African regions is presented in Figure 3A, and Sub-Saharan Africa region in Figure 3B. The regional comparison of the pooled small ruminant seroprevalence is presented in Figure 4.

## 4. Discussion

Brucellosis prevention and control programs in Africa face many challenges, including limited veterinary and healthcare infrastructures and facilities, increased mobility of pastoralist communities (including cross-border mobility) with their livestock in search of pasture and communal watering grounds, a lack of awareness and education on the risks associated with brucellosis or how to prevent it, traditional livestock management practices, and little knowledge about the prevalence of brucellosis [22,23,184,185,186]. This 26-year comprehensive review of epidemiological studies may provide important insights into the prevalence of small ruminant brucellosis in African countries, regions, and the entire continent, offering essential groundwork for planning brucellosis prevention and control programs. The findings of this study highlight the significant variations in the studies, as well as the prevalence of small ruminant brucellosis across African countries and regions.

A total of 28 African countries (more than half) from all 5 African regions and 151 qualified study articles for this study reported the presence of small ruminant brucellosis in Africa. Africa’s descriptive apparent pooled seroprevalence was 4.9% (95% CI: 3.0–7.5%, and pooled PCR positivity was 12.7% (95% CI: 6.0–21.9%), both with very wide prediction intervals. Even though the results are descriptive, they are a concerning issue of zoonotic infections, as well as economic losses. The reports of small ruminant brucellosis in 28 countries, and all African regions may also imply that brucellosis may also be prevalent in some the non-reporting countries. Pastoralists migrate seasonally from place to place in search of water and pasture [185]. The seasonal migration of pastoralists could also be cross-border, which can play a significant role in the transmission of brucellosis as a transboundary disease. As *Brucella* species including *B. melitensis* and *B. abortus* were also identified in wild animals in Africa [187], pastoralists may also encourage the free circulation of small ruminant brucellosis between domestic animals, and between wild and domestic animals, including transboundary circulations. The reason for not reporting could be that there may not be studies in these countries, or the studies from these countries may not be included in the databases used in this study. This gap underscores the need for standardized, multi-country comparative studies using uniform sampling frames and diagnostic protocols to reliably estimate the true prevalence of small ruminant brucellosis across Africa.

The study articles that reported small ruminant brucellosis were significantly unequally distributed across the reporting African countries, ranging from 46 articles from Ethiopia (the highest number) to the only one article from Botswana, Burkina Faso, Djibouti, Gambia, Ghana, Guinea, Morocco, and Niger (the lowest number). The crude total seroprevalence of small ruminant brucellosis in African countries is also significantly different, ranging from 19.53% in Libya (the highest prevalence), to 0.18% in Morocco (the lowest prevalence). The observed variations in estimates of the apparent prevalence of small ruminant brucellosis across African countries may be explained by several factors. First, true prevalence differences likely exist due to local husbandry practices and transboundary animal movements. Seasonal transhumance for pasture access has been identified as the main risk factor for brucellosis transmission between pastoral zones of Côte d’Ivoire and Mali [188]. The coexistence of nomadic pastoralism with settled intensive farming systems in Nigeria has created conditions conducive to disease emergence [189]. In Mali, seroprevalence varies significantly by production system, with peri-urban farms showing the highest burden (38.1%) compared to pastoral (24.3%) systems [167]. Second, there is considerable variability in diagnostic tests’ sensitivity and specificity. In Ethiopia, while RBT demonstrated 100% sensitivity in sheep and goats, false-positive results necessitated confirmation by ELISA [36]. In Kenya, the febrile *Brucella* antigen test showed only 76.5% sensitivity and 71.2% specificity compared to RBT, leading to potential misdiagnosis [190]. Third, there is an uneven number of representative articles per country, which may reflect differences in research investment, surveillance infrastructure, or publication bias. In Nigeria, which has the second-largest population of poor livestock keepers globally, available brucellosis evidence remains “scant and fragmented,” with most reports providing only serological evidence without species identification [189]. Furthermore, existing border surveillance mechanisms between West African countries fail to achieve coordinated transborder disease monitoring, limiting the comparability of prevalence estimates across nations [188]. Fourth, there is low and uneven vaccination coverage across Africa. This leads to a wide range of disease prevalence, as the implementation of effective and sustainable control programs has often failed in the worst-affected areas [191]. These variations and other factors underscore the need for standardized, multi-country comparative studies using uniform sampling frames and diagnostic protocols to reliably estimate the true prevalence of small ruminant brucellosis across Africa.

The distribution of the reported African countries is significantly different across their respective regions, ranging from nine countries in East Africa (the highest number), to only one country in Central Africa (the lowest number). The descriptive pooled seroprevalence of small ruminant brucellosis also varies across the regions, ranging from 6.2% (95% CI: 3.8–9.4%) in East Africa (the highest), to 3.3% (95% CI: 1.2–7.4%) in West Africa (the lowest). East Africa’s descriptive pooled seroprevalence of small ruminant brucellosis, 6.2% (95% CI: 3.8–9.4%), is comparatively lower than the seroprevalence of brucellosis in small ruminants in East Africa reported by Djangwani et al. [192], ranging from 0% to 20.0% among goats and 0% to 13.8% among sheep. The descriptive pooled seroprevalence of small ruminant brucellosis in North Africa, 4.90% (95% CI: 1.8–10.7%), is comparable to the reported prevalence of the small ruminant brucellosis in North Africa by Franc et al. [193], ranging from 0.1% to 7.5%. The pooled seroprevalence of small ruminant brucellosis in the Sub-Saharan Africa region, 3.60% (95% CI: 2.2–5.7%), is slightly lower than the reports by Ducrotoy et al. [194], varying between 0% and 4.8% for sheep and between 0% and 5.5% for goats.

Small ruminant brucellosis is reported using different diagnostic methods in Africa. Although the number of tested animals significantly differs among the brucellosis diagnostic methods, it is reported using all the brucellosis diagnostic methods, including serological tests (RBPT, CFT, ELISA, STAT, NHIP, BAPAT, MRT, LFA, 2-ME, and FPA), molecular techniques (PCR and WGS), and *Brucella* isolation methods (BSI, BCI, MZN). The crude total prevalence of brucellosis in small ruminants significantly varies across the utilized tests, ranging from 21.54% using WGS (the highest result) to 3.37% using 2-ME (the lowest result) (Table 2). The observed differences in brucellosis prevalence among the included studies arise from several factors, including diagnostic and design factors. First, serological tests differ in their diagnostic accuracy. RBPT has high sensitivity but variable specificity, whereas ELISA methods generally offer higher specificity. According to the OIE Manual, RBPT is recommended as a screening test due to its high sensitivity; however, positive results require confirmation by more specific tests such as c-ELISA or CFT [195]. Second, most studies employ a serial testing strategy: testing only RBPT-positive animals with a confirmatory test. This approach increases specificity but reduces overall sensitivity compared to testing all animals with both methods, potentially underestimating true prevalence [195]. Third, false positives occur due to cross-reacting antibodies from other Gram-negative bacteria or prior vaccination, which is an inherent limitation of serological tests [14,195,196]. Fourth, PCR and culture measure biologically distinct states from serology. PCR can detect DNA when cultures remain negative, while serology reflects past exposure rather than active infection [197]. Fifth, sampling frames vary and dictate the study’s epidemiological validity. Randomized population designs yield unbiased prevalence estimates. Conversely, convenience sampling (abattoirs, outbreaks) introduces bias but maximizes cost-effectiveness and target detection in high-risk groups [198]. Therefore, without accounting for these diagnostic and sampling differences, direct comparisons of prevalence across studies are methodologically invalid.

The few molecular and bacteriological reports of this study confirm that both *B. melitensis* and *B. abortus* circulate across most of Africa (East, North, West, Southern, and Sub-Saharan regions), except Central Africa, where only *B. abortus* is found. However, species dominance varies by region: *B. melitensis* dominates in Eastern, Northern, and Southern Africa, while *B. abortus* dominates in Western and Central Africa. This spatial pattern is shaped by host species, livestock systems, and historical ecological factors. First, the predominance of *B. melitensis* in North Africa aligns with the region’s extensive preferred host: small ruminant (sheep and goats) production systems. In contrast, West Africa’s predominance of *B. abortus* corresponds to the region’s greater reliance on cattle husbandry [188]. East Africa presents an interesting hybrid model where both species circulate significantly, likely due to the region’s diverse livestock systems that integrate cattle, goats, sheep, and camels. In Tanzania, molecular surveillance of dairy cattle revealed that *B. melitensis* was the predominant species detected (66.2%), suggesting spillover from small ruminant reservoirs [199]. These findings may indicate that East Africa serves as a transition zone where livestock movement and mixed husbandry practices facilitate cross-species transmission. Second, *B. melitensis* dominates in arid/semi-arid regions (North, East, Southern Africa), where small ruminants are better adapted, while *B. abortus* dominates in humid zones (West, Central Africa), where cattle farming is more viable [200]. Wildlife–livestock interfaces also help maintain brucellosis strains. *B. melitensis* found in sable antelope in South Africa formed a unique African sub-clade in phylogenetic analyses [201]. Third, *B. melitensis* poses a greater human health threat than *B. abortus*, causing more severe disease and treatment challenges [202]. High-risk regions (Eastern, Northern, Southern Africa) where *B. melitensis* predominates face the greatest zoonotic threat. Livestock mobility amplifies the zoonotic risk; cross-border transhumance introduces *Brucella* to naive populations, and informal animal markets create untraceable transmission chains [203]. A few references reported the predominant endemicity of brucellosis due to *B. melitensis* (mainly from goats and sheep), followed by *B. abortus* (isolated from natural and non-specific hosts) in the North Africa region [204,205], supporting this result. As the cultural practices of pastoralists in Africa include consuming raw animal products (meat and milk), home-based slaughtering practices without adequate hygiene measures, assisting in the birthing process of animals without using protective equipment, and reliance on ethno-veterinary knowledge [185], there is a high risk of zoonotic brucellosis infections.

Given Africa’s diverse economic conditions, production systems, veterinary infrastructure, and policy environments [200], a single set of prevention measures is impractical. We, therefore, propose a regionally stratified, tiered, and resource-aligned framework. First, in these extensive systems, livestock mobility, poor access to veterinary services, and limited cold-chain infrastructure make test-and-slaughter programs unfeasible and culturally unacceptable [194]. Therefore, mass vaccination of small ruminants, combined with public awareness campaigns on raw milk and cheese consumption, is the most feasible approach. In addition, given economic constraints, external aid (e.g., via the Global Framework for the Progressive Control of Transboundary Animal Diseases—GF-TADs) is essential to subsidize vaccines. Second, in areas where animals move across borders, reinfection is persistent, making single-country approaches ineffective. Therefore, coordinated bilateral or regional vaccination campaigns are necessary. The WOAH’s Performance of Veterinary Services (PVS) Pathway recommends joint vaccination schedules, shared cold-chain facilities at border markets, and cross-border disease surveillance committees [206]. Third, in settings with strong veterinary capacity, cold chains, and market-oriented production, a test-and-slaughter or test-and-segregation strategy is cost-effective. Key measures include serological testing of replacement animals, pasteurizing waste milk fed to kids and lambs, and controlling the movement of seropositive herds, which can result in the elimination of the zoonotic disease within two to three years [207]. This study also recommends a tiered, WOAH-aligned surveillance algorithm: all countries should use RBPT or ELISA for screening; countries with confirmation capacity should add CFT or ELISA (competitive or indirect ELISAs); PCR and bacterial culture should be reserved for regional reference laboratories and outbreak investigations [208,209], with the need for integrated One Health surveillance and control strategies.

There are several limitations recognized in this review study. The first limitation is that there is a lack of equally distributed references for all African countries. The referenced articles are significantly unequally distributed across the reporting African countries, including some articles reporting only a specific place (not country aggregation), which may not be representative of the countries. The second limitation is diagnostic uncertainty. As most of the apparent prevalence of brucellosis in small ruminants in African countries is reported using serological methods, there is a probability of including false positive results, as serological tests cannot Differentiate Infected from Vaccinated Animals (DIVA), and they also cannot differentiate other infections that produce similar antibodies, like Yersinia enterocolitica O:9 and a few others. Furthermore, there are no statistics to inform whether the tested animals have a history of vaccination or not. As we totaled the serological test results, the total number may also include double counting of animals due to possible duplicate test results. The third and final limitation is that the limited selection of databases (PubMed, ScienceDirect, and Google Scholar) may not be the most relevant databases for veterinary, agricultural, and African regional literature. In addition, the search method was a title-only search, excluded negative results, applied an English-restricted search, and lacked study-level code/data. Therefore, despite the extensive searches in the three utilized databases, there is a probability that not all the study articles reporting the prevalence of brucellosis in small ruminants in African countries have been gathered. Some articles that report the presence of brucellosis in small ruminants may not include the search terms in their titles, and articles available outside of the three employed databases have a high probability of being omitted from inclusion in this study.

## 5. Conclusions

Small ruminants hold unquestionable economic, social, and cultural importance in Africa. To date, no continent-wide prevalence study exists for brucellosis in small ruminants. However, available studies from several African countries provide apparent serological and molecular evidence of *Brucella* exposure (seroprevalence) and infection (molecular detection) in small ruminant populations. Both *B. abortus* and *B. melitensis* have been identified via molecular techniques and isolation methods in some of these reporting countries where such testing was performed.

Nevertheless, because this systematic review and meta-analysis revealed high heterogeneity (*I*^2^ > 90%), wide prediction intervals, and substantial diagnostic variability across included studies (countries), we cannot provide a precise estimate of continental endemic prevalence, nor can we definitively state that brucellosis is endemic in all African regions or across the entire continent. Instead, the evidence indicates that brucellosis is present in many (but not all) African countries and regions, with marked local and national variations.

Given these limitations, we refrain from making broad policy recommendations (e.g., cooperative continental programs, enforcement of control strategies) as direct conclusions from this meta-analysis. Instead, future research should prioritize standardized, region-specific surveys using validated diagnostic tests (e.g., i-ELISA with confirmatory culture or PCR) to enable robust comparisons. Policy efforts to safeguard the role of small ruminants should initially focus on local, evidence-based interventions, acknowledging the substantial heterogeneity observed across Africa.

## Figures and Tables

**Table 1:** Prevalence of small ruminants’ brucellosis in Ethiopia, Nigeria, Egypt, Uganda, Tanzania, and Kenya using the most common diagnostic methods used in African countries, including RBPT, CFT, ELISA, the Serum Tube Agglutination Test (STAT), and PCR.

S. No.	Countries	Pooled Prevalence of Small Ruminants’ Brucellosis by Tests					References
		RBPT	CFT	ELISA	STAT	PCR	
1	Ethiopia	1983/36,593 (5.42%)	1068/30,902 (3.46%)	1285/15,585 (8.25%)		107/935 (11.44%)	[23,32,33,35,36,37,38,39,40,41,42,43,44,45,46,47,48,49,50,51,52,53,54,55,56,57,58,59,60,61,62,63,64,65,66,67,68,69,70,71,72,73,74,75,76]
2	Nigeria	1235/9811 (12.59%)		228/4165 (5.47%)	419/4868 (8.61%)	13/119 (10.92%)	[77,78,79,80,81,82,83,84,85,86,87,88,89,90,91,92,93]
3	Egypt	287/3425 (8.38%)	936/55044 (1.70%)	31/360 (8.61%)	78/1256 (6.21%)	53/53 (100%)	[94,95,96,97,98,99,100,101,102,103,104,105,106,107]
4	Uganda	307/4855 (6.32%)		41/712 (5.76%)	141/1446 (9.75%)		[108,109,110,111,112,113,114,115,116]
5	Tanzania	291/10,473 (2.78%)		401/10,758 (3.73%)		25/120 (20.83%)	[7,117,118,119,120,121,122,123]
6	Kenya	46/296 (15.54%)		676/4941 (13.68%)		136/639 (21.28%)	[124,125,126,127,128,129,130]

**Table 2:** Total test result records of brucellosis in small ruminants across the entire African continent using various diagnostic methods.

S. No.	Tests	Total Number of Tested Animals	Number of Positive Animals	Positive Rates (%)
1	RBPT	110,945	4996	4.50
2	CFT	237,939	10,791	4.54
3	ELISA	50,233	3749	7.46
4	PCR	7893	534	6.77
5	STAT	7570	638	8.43
6	NHIP	925	51	5.51
7	BAPAT	1789	105	5.87
8	MRT	718	55	7.66
9	LFA	1320	165	12.50
10	2-ME	415	14	3.37
11	FPA	188	22	11.70
12	MZN	319	53	16.61
13	WGS	390	84	21.54
14	BSI	2382	153	6.42
15	BCI	28	5	17.86
	Total	423,054	21,415	5.06

**Table 3:** Crude prevalence of brucellosis in small ruminants in the Sub-Saharan African region using various diagnostic methods.

S. No.	Tests	Total Number of Tested Animals	Number of Positive Animals	Positive Rates (%)
1	RBPT	100,628	4481	4.45
2	CFT	125,853	2177	1.73
3	ELISA	46,472	3111	6.69
4	PCR	6988	381	5.45
5	STAT	6314	560	8.87
6	NHIP	925	51	5.51
7	MRT	108	14	12.96
8	LFA	1320	165	12.50
9	2-ME	415	14	3.37
10	FPA	188	22	11.70
11	MZN	319	53	16.61
12	WGS	295	43	14.58
13	BSI	2238	119	5.32
	Total	292,091	11,196	3.83

## Data Availability

No new data were created or analyzed in this study. Data sharing is not applicable to this article.

## Supplementary Materials

The following supporting information can be downloaded at https://www.mdpi.com/article/10.3390/vetsci13070638/s1, Supplementary Tables S1: Leave-one-out sensitivity analysis for regional pooled small ruminant brucellosis seroprevalence (East Africa and Sub-Saharan Africa regions); Supplementary Table S2: Comparison of transformation methods. Data: 27 African countries, small ruminant brucellosis seroprevalence; Supplementary Table S3: Results of the robustness analysis (for the full Africa dataset, 27 countries).

## Abbreviations

The following abbreviations are used in this manuscript:

2-ME	2-Mercaptoethanol
BSI	Bacterial Strain Isolation
USD	United States Dollar
PRISMA	Preferred Reporting Items for Systematic Reviews and Meta-analyses
JBI	Joanna Briggs Institute
RBPT	Rose Bengal Plate Test
CFT	Complement Fixation Test
ELISA	Enzyme-Linked Immunosorbent Assay
PCR	Polymerase Chain Reaction
WGS	Whole-Genome Sequencing
STAT	Serum Tube Agglutination Test
LFA	Lateral Flow Assay
MRT	Milk Ring Test
BCI	*Brucella* Cultural Isolation
BAPAT	Buffered Acidified Plate Antigen Test
NHIP	Native Hapten Immunoprecipitation
FPA	Fluorescence Polarization Assay
MZN	Modified Ziehl–Neelsen Staining
ISO	International Organization for Standardization
DIVA	Differentiate Infected from Vaccinated Animals
WOAH	World Organisation for Animal Health
PVS	Performance of Veterinary Services
GF-TADs	Global Framework for the Progressive Control of Transboundary Animal Diseases
