# Supplementary material for: Prevalence of Brucellosis in Small Ruminants in Africa from 2000 to 2025: A Systematic Review and Meta-Analysis
Source: Vet Sci. 2026 Jun 30;13(7):638. doi: 10.3390/vetsci13070638 (PMC13431580; doi:10.3390/vetsci13070638)
Supplement: Supplementary file 1 [file vetsci-13-00638-s001.zip › vetsci-4300918-supplementary.pdf]

Supplementary Tables

Supplementary Tables S1. Leave-one-out sensitivity analysis for regional pooled small ruminant brucellosis seroprevalence (East Africa and Sub-Saharan Africa regions).

Excluded country	Pooled prevalence without excluded (%)	Difference from original (percentage points)
Ethiopia	6.0	-0.2
Uganda	6.1	-0.1
Kenya	5.4	-0.8
Tanzania	6.3	+0.1
Eritrea	6.2	0.0
Somalia	6.2	0.0
Rwanda	6.0	-0.2
South Sudan	6.1	-0.1
Djibouti	6.2	0.0

* East Africa (9 countries; original pooled seroprevalence = 6.2%).

Excluded country	Pooled prevalence without excluded (%)	Difference from original (percentage points)
Ethiopia	3.6	-0.1
Uganda	3.6	-0.1
Kenya	3.5	-0.2
Nigeria	3.5	-0.2
Tanzania	3.7	0.0
Eritrea	3.7	0.0
Sudan	3.7	0.0
Somalia	3.7	0.0
Burkina Faso	3.7	0.0
Niger	3.7	0.0
South Africa	3.8	+0.1
Ghana	3.6	-0.1
Mali	3.7	0.0
Gambia	3.7	0.0
Cote d'Ivoire	3.7	0.0
Rwanda	3.6	-0.1
Namibia	3.8	+0.1
South Sudan	3.6	-0.1
Djibouti	3.7	0.0
Guinea	3.7	0.0
Zambia	3.6	-0.1
Cameroon	3.7	0.0

* Sub-Saharan Africa (22 countries; original pooled prevalence = 3.6%)

Supplementary Table S2. Comparison of transformation methods. Data: 27 African countries, small ruminant brucellosis seroprevalence.

Method	Transformation/ Model	Pooled prevalence (%)	95% CI (%)	Heterogeneity I^2 (%)
Freeman-Tukey double arcsine (original)	Variance-stabilizing	4.93	3.03 – 7.46	99.5%
Logit transformation	Log(odds)	5.01	2.90 – 7.79	99.5%
Binomial-normal GLMM	No transformation (exact binomial)	4.92	3.12 – 7.42	99.5%

Supplementary Table S3. Results of the robustness analysis (for the full Africa dataset, 27 countries).

Estimator	Pooled prevalence (%)	95% CI (%)	Heterogeneity I^2 (%)
DerSimonian-Laird (original)	4.9	3.0 – 7.5	99.5
REML + Hartung-Knapp	5.1	2.8 – 8.1	99.5
Paule-Mandel + Hartung-Knapp	5.0	2.9 – 7.9	99.5
